# The positive relationship between androgen receptor splice variant-7 expression and the risk of castration-resistant prostate cancer: A cumulative analysis

**DOI:** 10.3389/fonc.2023.1053111

**Published:** 2023-02-14

**Authors:** Shankun Zhao, Jian Liao, Shilong Zhang, Maolei Shen, Xin Li, Libo Zhou

**Affiliations:** ^1^ Department of Urology, Taizhou Central Hospital (Taizhou University Hospital), Taizhou, Zhejiang, China; ^2^ Department of Nephrology, Jiaxing Hospital of Traditional Chinese Medicine, Jiaxing, Zhejiang, China; ^3^ Clinical Medical College, Zhejiang Chinese Medical University, Hangzhou, Zhejiang, China; ^4^ Department of Urology, The First Affiliated Hospital of Nanchang University, Nanchang, China

**Keywords:** androgen receptor variant-7, castration-resistant prostate cancer, systematic review, cumulative analysis, expression

## Abstract

**Background:**

At present, androgen deprivation therapy (ADT) is still the standard regimen for patients with metastatic and locally advanced prostate cancer (PCa). The level of androgen receptor splice variant-7 (AR-V7) in men with castration-resistant prostate cancer (CRPC) has been reported to be elevated compared with that in patients diagnosed with hormone-sensitive prostate cancer (HSPC).

**Aim:**

Herein, we performed a systematic review and cumulative analysis to evaluate whether the expression of AR-V7 was significantly higher in patients with CRPC than in HSPC patients.

**Methods:**

The commonly used databases were searched to identify the potential studies reporting the level of AR-V7 in CRPC and HSPC patients. The association between CRPC and the positive expression of AR-V7 was pooled by using the relative risk (RR) with the corresponding 95% confidence intervals (CIs) under a random-effects model. For detecting the potential bias and the heterogeneity of the included studies, sensitivity analysis and subgroup analysis were performed. Publication bias was assessed Egger’s and Begg’s tests. This study was registered on PROSPERO (ID: CRD42022297014).

**Results:**

This cumulative analysis included 672 participants from seven clinical trials. The study group contained 354 CRPC patients, while the other group contained 318 HSPC patients. Pooled results from the seven eligible studies showed that the expression of positive AR-V7 was significantly higher in men with CRPC compared to those with HSPC (RR = 7.55, 95% CI: 4.61–12.35, *p* < 0.001). In the sensitivity analysis, the combined RRs did not change substantially, ranging from 6.85 (95% CI: 4.16–11.27, *p* < 0.001) to 9.84 (95% CI: 5.13–18.87, *p* < 0.001). In the subgroup analysis, a stronger association was detected in RNA *in situ* hybridization (RISH) measurement in American patients, and those studies were published before 2011 (all *p* < 0.001). There was no significant publication bias identified in our study.

**Conclusion:**

Evidence from the seven eligible studies demonstrated that patients with CRPC had a significantly elevated positive expression of AR-V7. More investigations are still warranted to clarify the association between CRPC and AR-V7 testing.

**Systematic review registration:**

https://www.crd.york.ac.uk/prospero/, identifier CRD42022297014.

## Introduction

According to the data from Cancer Statistics 2022, prostate cancer (PCa) is the most frequently diagnosed male cancer in Western countries (1, 2). Moreover, PCa is one of the leading causes of cancer mortality in developed countries (1, 2). The growth and differentiation of normal prostate cells depend on androgens for the activation of androgen receptors (ARs) (3). Also, androgens play an essential role during all phases of the growth of PCa cells (4). AR signaling is the foundation for the proliferation and survival of PCa cells. A human AR gene can be found on chromosome Xq11-12. It is composed of eight exons encoding a 110-kDa protein. Structurally, the human AR protein is composed of an N-terminal transactivation domain, a hinge region, a central DNA-binding domain, and a C-terminal ligand-binding domain (5). The binding of androgen to the AR ligand-binding domain (LBD) allows the ligand-bound receptor to enter the nucleus and regulate androgen-responsive genes in the nucleus (6). At present, androgen deprivation therapy (ADT) is still the mainstay therapy for metastatic and advanced PCa. To a great extent, men with advanced PCa may initially respond to ADT, termed hormone-sensitive prostate cancer (HSPC). Unfortunately, the majority of patients may experience progression to castration-resistant prostate cancer (CRPC) within a median time frame of 24 to 36 months, although the levels of androgens continue to be low (7).

The current evidence suggests that CRPC may be not independent of the effect of androgen, but AR signaling continues to be essential (8). The researchers found that CRPC could express not only AR but also the androgen-responsive genes. The AR signaling pathway is still functional when the androgen is depleted (9). The AR axis still plays a role in CRPC and is accountable for the progression of the disease (10), and a new generation of AR-directed agents have recently emerged, i.e., androgen biosynthesis inhibitor “abiraterone” (11) and the AR antagonist “enzalutamide” (12). The mechanisms of ablation-resistant AR-mediated signaling pathways have not yet been fully elucidated. The mechanisms by which AR are activated in CRPC have been hypothesized to be different, including mutation and augmentation of AR gene (13, 14). AR may turn out to be more susceptible to stimulation by androgens or other ligands due to intratumoral synthesis of androgens, as well as epigenetic and genetic alterations (15–17). Moreover, the androgen receptor splice variants (AR-Vs) are frequently expressed in CRPC (18, 19). To date, more than 20 AR-Vs were identified, and AR-V7 (also named AR3) is one of the most clinically significant variants (20).

AR-V7 has been identified as one of the leading splice variants in both localized and advanced PCa (21). AR-V7 is deficient with the ligand-binding domain (androgen-binding site) but retains the transactivating N-terminal domain. Since AR-V7 serves as an important transcription factor, it constitutively activates and promotes the activation of its target genes (22). Several clinical trials have reported that the AR-V7 level was significantly greater in CRPC when compared with HSPC (23–25). According to these studies, a trend toward a higher level of AR-V7 was observed in patients with CRPC.

Since a number of studies have identified the potential relationship between CRPC and AR-V7, it is clinically meaningful to summarize the evidence on this issue by conducting a meta-analysis. In this study, we performed a cumulative study to summarize and analyze the evidence on the association between CRPC and the AR-V7 expression.

## Methods

The current cumulative analysis was conducted according to the guidelines of Preferred Reporting Items for Systematic Reviews and Meta-Analyses (PRISMA) (26), which was registered on the PROSPERO (ID: CRD42022297014).

### Search strategy

The Medline, Cochrane Library, and Embase were searched for systematic literature reviews. The time frame for searching the eligible studies was up to April 2022. Searches were conducted by using the subject headings and keywords. The following search terms were used: ((((((“Prostatic Neoplasms”[Mesh]) OR (Prostate Cancer)) OR (Prostatic Cancer)) AND ((((AR-V7) OR AR3) OR receptor splicing variant 7) OR androgen receptor 3))) AND castration-resistant prostate cancer). Moreover, we further reviewed the reference lists of the relevant articles to detect more eligible studies. Participants and the language of the search were restricted to American patients and English, respectively.

### Quantification of AR-V7

Clinical trials in which AR-V7 was investigated by any of the existing instruments were considered to be eligible. These included immunohistochemical (IHC) staining analysis, RNA *in situ* hybridization (ISH), PCR, and protein analysis.

### Study selection

Study eligibility was fully determined by the PICOS criteria, namely, the patient population, intervention or exposure, comparison, and outcome (PICOS) study design. The inclusion criteria included the following: 1) participants: men with prostate cancer PCa. 2) Interventions: CRPC. 3) Comparisons: HSPC. 4) Outcomes: the positive expression of AR. 5) Study design: any study designs. Furthermore, additional studies included in this review were supposed to provide the relative risk (RR) estimates with the corresponding 95% confidence intervals (CIs). A list of exclusion criteria was provided, as follows: 1) no control data; 2) updated or duplicated data; 3) review articles; 4) meeting abstract, commentaries, editorials, congress reports, letters, or case reports; and 5) animal or *in vitro* experiments.

### Quality assessment and data extraction

According to the predetermined selection criteria, two authors independently extracted the data. Data were obtained from the included studies, as follows: names of the first author, study design, publication year, study areas, the sample sizes of the study group and the control group, methods of AR-V7 detection, and the effect measures (HR, RR, or OR) with their 95% CI. The quality of cross-sectional studies was assessed by using the quality methodology checklist (27). The Newcastle–Ottawa Scale was applied to evaluate the study quality of the case–control/cohort studies (28).

### Statistical analyses

The strength of the association between CRPC and AR expression was assessed by the pooled RRs and 95% CIs. Results with *p*-values <0.05 were defined as statistically significant. Heterogeneity across studies was determined by using the *I* ^2^ statistic and Cochran’s Q statistic (29). Fixed-effect models were used when there was no significant heterogeneity (*I* ^2^ < 50%, *p* > 0.10). Otherwise, a random-effects model was applied. Moreover, an analysis of sensitivity was conducted by omitting one study at one time to assess how it affected the overall risk estimate. The origins of heterogeneity were further examined by subgroup analyses. Publication biases were determined by using Begg’s and Egger’s tests (30, 31). *p* > 0.05 indicated no publication bias, while *p* < 0.05 was judged to be a statistical publication bias. All the analyses presented within the present study were conducted the Stata 12.0 software (Stata Corp LP, College Station, TX, USA).

## Results

### Results from the literature search

**Figure 1:**
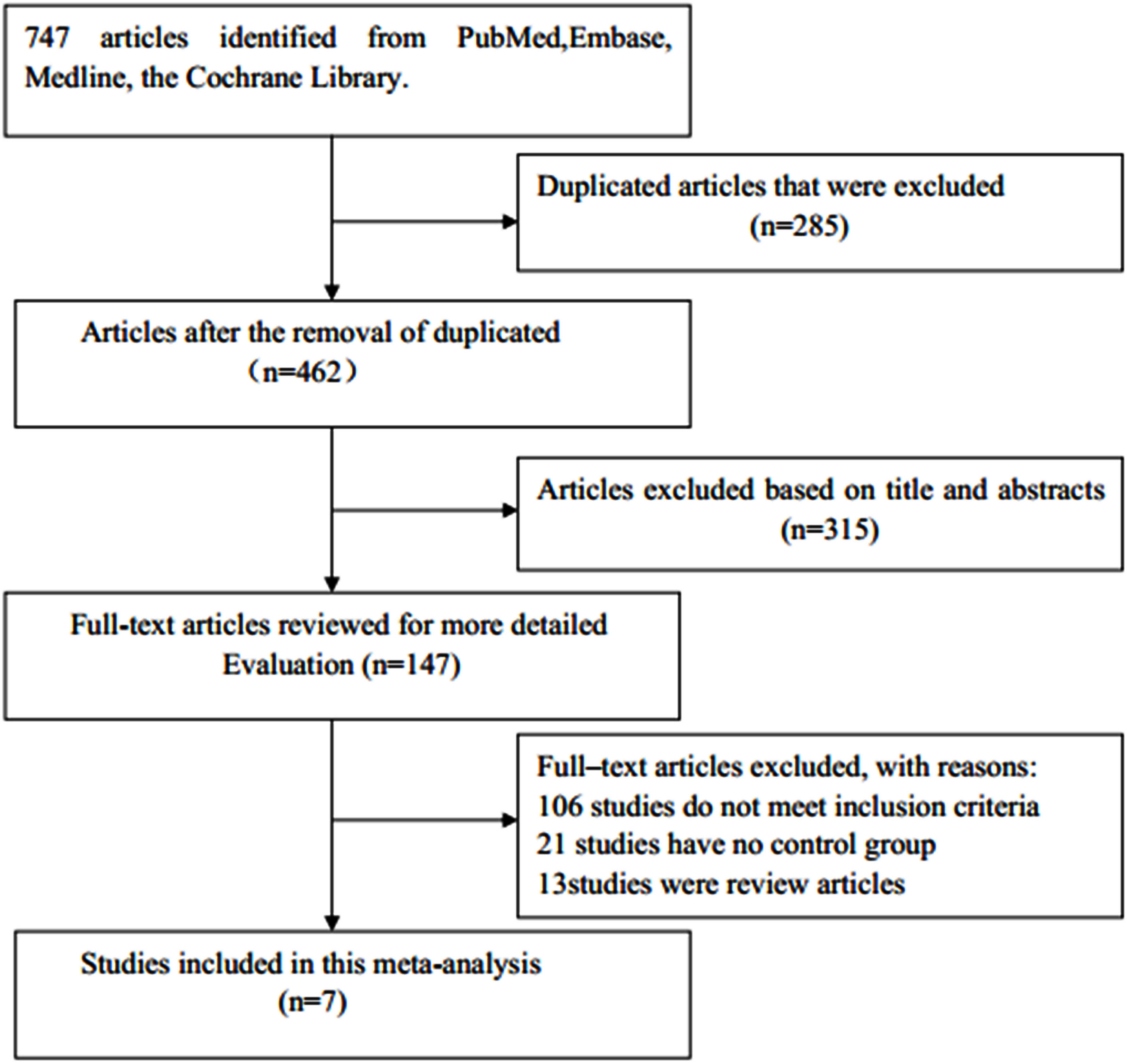
Flowchart of study selection.

A diagram of the study selection process is shown in **Figure 1**. A total number of 747 articles were identified in the initial search, of which 285 duplicates were eliminated. Of the remaining articles, after the titles and abstracts were read, 315 articles were excluded. A total of 147 potentially relevant studies remained for further review. Among them, 106 studies were eliminated for not meeting the inclusion criteria, 21 for having no control group, and 13 for being reviews. Finally, seven observational studies were included in this meta-analysis (23–25, 32–35).

### Study characteristics

The characteristics of eligible publications are listed in **Table 1**. The study design of all included studies was cross-sectional. All of the eligible publications were published from 2009 to 2017. Among the seven included studies, a total of 672 participants were enrolled, 354 of whom were CRPC patients, while the remaining 318 participants were HSPC patients (the sample sizes ranged from 9 to 162). The assessment of AR-V7 expression was inconsistent among studies.

**Table 1:** Characteristics of the included studies in the meta-analysis.

Study	Year	Country	Study design	AR-V7 detection	Hormone-sensitive prostate cancer		Castration-resistant prostate cancer		RR (95% CI)
					Patients	Age	Patients	Age	
Welti et al.	2016	UK	Cross-sectional	IHC	7/33	NA	25/35	67.5 (64.2–75.3)	9.29 (3.06–28.20)
Zhang et al.	2011	USA	Cross-sectional	IHC	2/50	NA	39/162	NA	7.61 (1.77–32.75)
Saylor et al.	2016	USA	Cross-sectional	RISH	1/30	NA	12/12	NA	491.67 (18.72–12913.73)
Qu et al.	2015	China	Cross-sectional	IHC	22/104	70 (43–84)	27/46	65 (50–79)	5.30 (2.50–11.24)
Hornberg et al.	2011	Sweden	Cross-sectional	IHC	8/10	79 (60–85)	30/30	70 (51–86)	17.94 (0.78–410.42)
Hu et al.	2009	USA	Cross-sectional	PCR and protein analysis	34/82	LNA	21/25	NA	7.41 (2.33–23.55)
Zhu et al.	2017	USA and UK	Cross-sectional	RISH	0/9	NA	15/44	NA	6.89 (0.45–105.79)

IHC, immunohistochemistry; RISH, RNA in situ hybridization; NA not available; RR, relative risk.

### Study quality

**Supplementary Table 1** summarizes the quality assessment results for the cross-sectional studies.

### Synthesis of results

**Figure 2:**
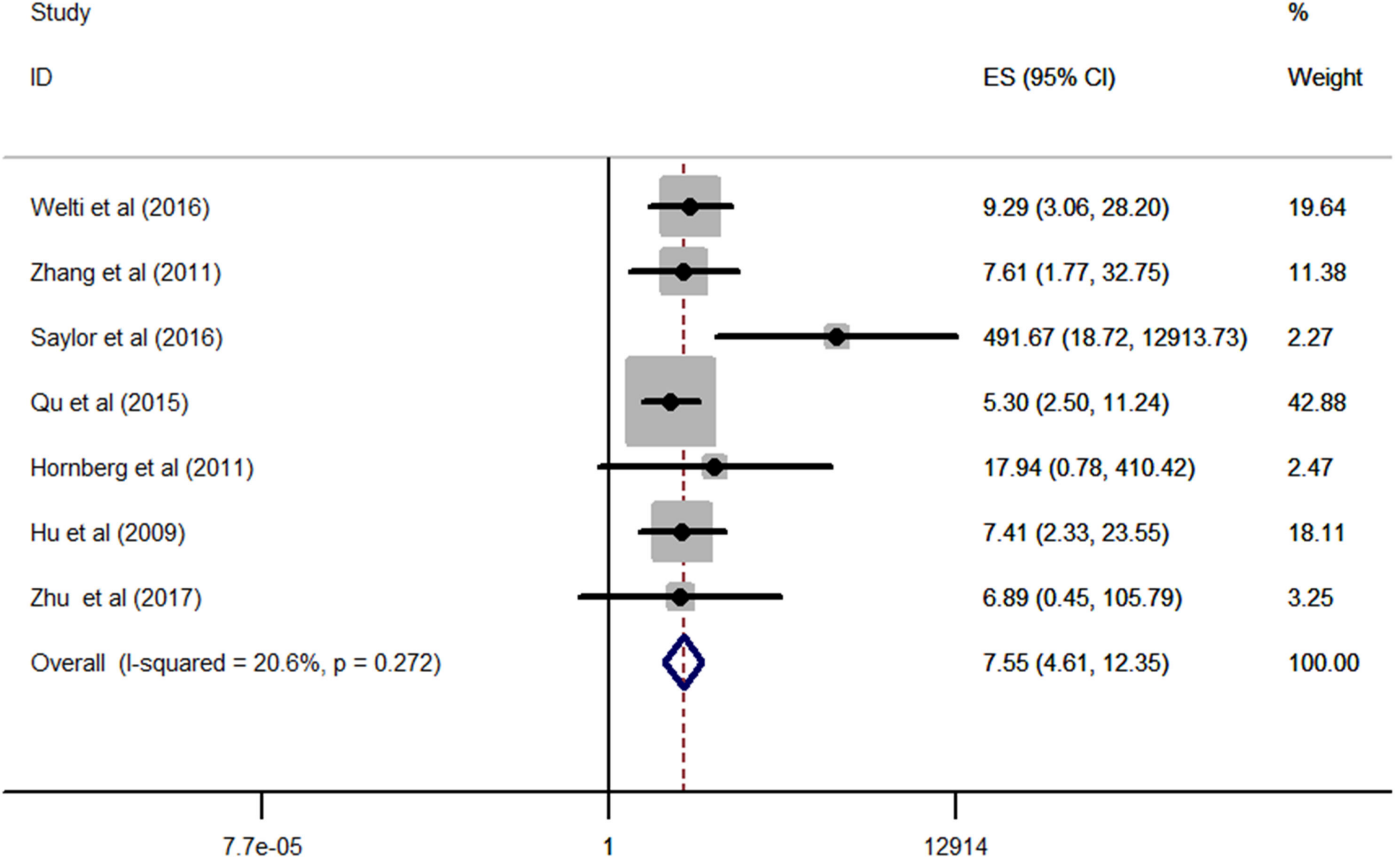
Forest plots of meta-analysis of the included studies on the association between CRPC and positive expression of AR-V7. CRPC, castration-resistant prostate cancer.

No significant statistical heterogeneity was detected among all the eligible studies (*I* ^2^ = 20.6%, *p* = 0.272); thus, the fixed-effects model was conducted to pool the data. As displayed in **Figure 2**, the combined results revealed that patients with CRPC had a significantly higher expression of AR-V7 than the individuals with HSPC (RR = 7.55, 95% CI: 4.61–12.35, *p* < 0.001), which showed that CRPC was strongly correlated with an increased positive expression of AR-V7.

### Sensitivity analysis

**Figure 3:**
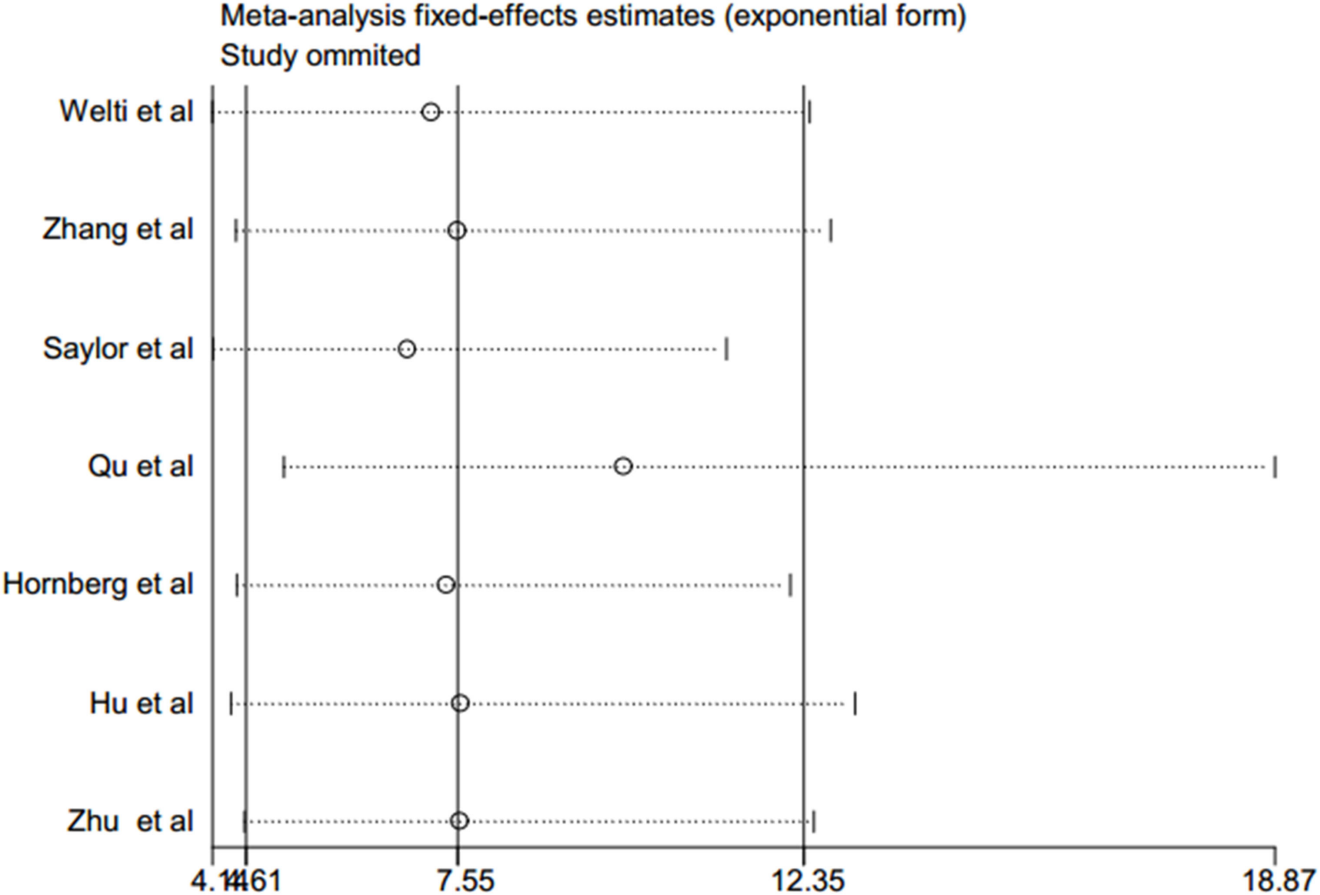
Sensitivity analysis after each study was excluded by turns.

A sensitivity analysis of this meta-analysis was conducted to evaluate its reliability with regard to the association between CRPC and positive expression of AR-V7. Each of the individual studies was excluded in turn to recalculate the synthesized RR. The overall pooled return on investment did not change substantially, with a range from 6.85 (95% CI: 4.16–11.27, *p* < 0.001) to 9.84 (95% CI: 5.13–18.87, *p* < 0.001) after eliminating any one of the included studies (**Table 2** and **Figure 3**). These results suggested that no single study dominated the combined RR, which strengthened the evidence of this study.

**Table 2:** Sensitivity analysis after each study was excluded by turns.

Study omitted	RR (95% CI) for remainders	Heterogeneity	
		*I* ^2^ (%)	*p*
Welti et al., 2016	7.17 (4.14, 12.42)	32.3	0.19
Zhang et al., 2011	7.54 (4, 47, 12.72)	33.8	0.18
Saylor et al., 2016	6.85 (4.16, 11.27)	0	0.95
Qu et al., 2015	9.84 (5.13, 18.87)	17.6	0.30
Hornberg et al., 2011	7.38 (4.49, 12.15)	31.1	0.20
Hu et al., 2009	7.58 (4.40, 13.05)	33.8	0.18
Zhu et al., 2017	7.57 (4.59, 12.49)	33.8	0.18

RR, relative risk.

### Subgroup analyses

To further obtain the potential relationship between CRPC and the positive expression of AR-V7, subgroup analyses were performed according to the methods of AR-V7 detection, the geographical region, and the publication year (**Table 3**). In the subgroup analysis according to the methods of AR-V7 detection, a stronger association was detected in the RNA *in situ* hybridization (RISH) group (RR = 39.81, 95% CI: 4.90–323.51, *p* < 0.001) compared with the IHC group (RR = 6.72, 95% CI: 3.83–11.81, *p* < 0.001) and PCR and protein analysis group (RR = 7.41, 95% CI: 2.33–23.56, *p* < 0.001). Moreover, in the subgroup analysis according to the geographical region, a statistically significant association between CRPC and positive expression of AR-V7 could be observed in the UK, the USA, and China, with RR (95% CIs) of 9.29 (3.06–28.20, *p* < 0.001), 10.09 (4.21–24.18, *p* < 0.001), and 5.30 (2.50–11.24, *p* < 0.001), respectively. However, a similar association was not identified in Sweden (RR = 17.94, 95% CI: 0.78–411.52, *p* > 0.05). Finally, when further stratified by publication years 2009–2011 and 2012–2017, the RR of CRPC associated with positive AR-V7 expression was 8.00 (95% CI: 3.35–19.13, *p* < 0.001) and 7.34 (95% CI: 4.04–13.33, *p* < 0.001), respectively.

**Table 3:** Subgroup analysis of the association between CRPC and positive expression of AR-V7.

Study or subgroup		No. of studies	Heterogeneity		RR (95% Cl)	*p*
			*I* ^2^ (%)	*p*		
AR-V7 detection	IHC	4	0	0.78	6.72 (3.83, 11.81)	<0.001
	RISH	2	74.1	0.05	39.81 (4.90, 323.51)	<0.001
PCR and protein analysis	1	–	–	7.41 (2.33, 23.56)	–
Country	UK	1	–	–	9.29 (3.06, 28.20)	–
	USA	3	65.8	0.054	10.09 (4.21, 24.18)	<0.001
	China	1	–	–	5.30 (2.50, 11.24)	–
Sweden	1	–	–	17.94 (0.78, 411.52)	–
Year	≤2011	3	0	0.87	8.00 (3.35, 19.13)	<0.001
	>2011	4	28.6	0.064	7.34 (4.04, 13.33)	<0.001

IHC, immunohistochemistry; RISH, RNA in situ hybridization; RR, relative risk.

### Publication bias

**Figure 4:**
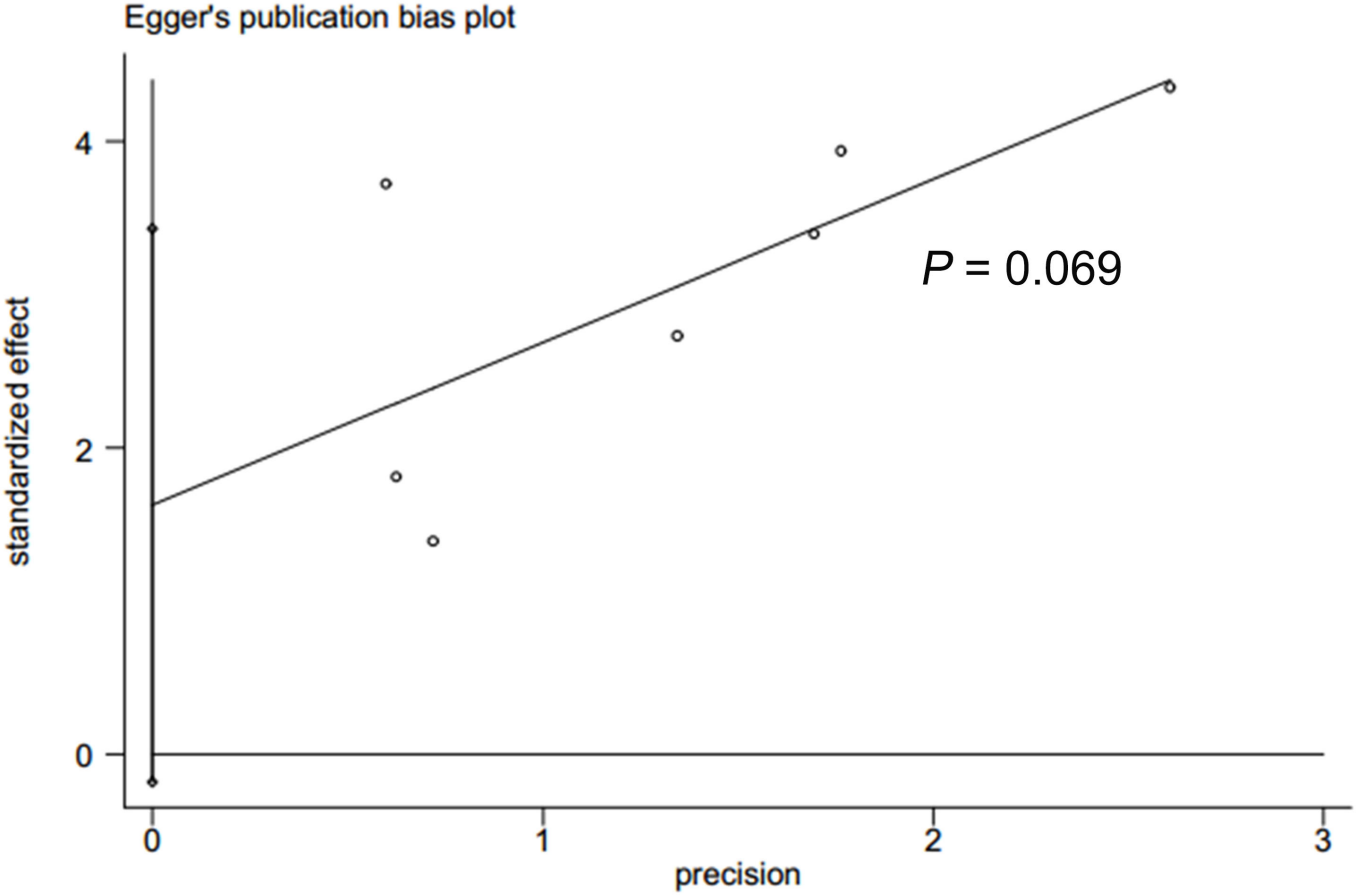
Eggers test to detect publication bias.

**Figure 5:**
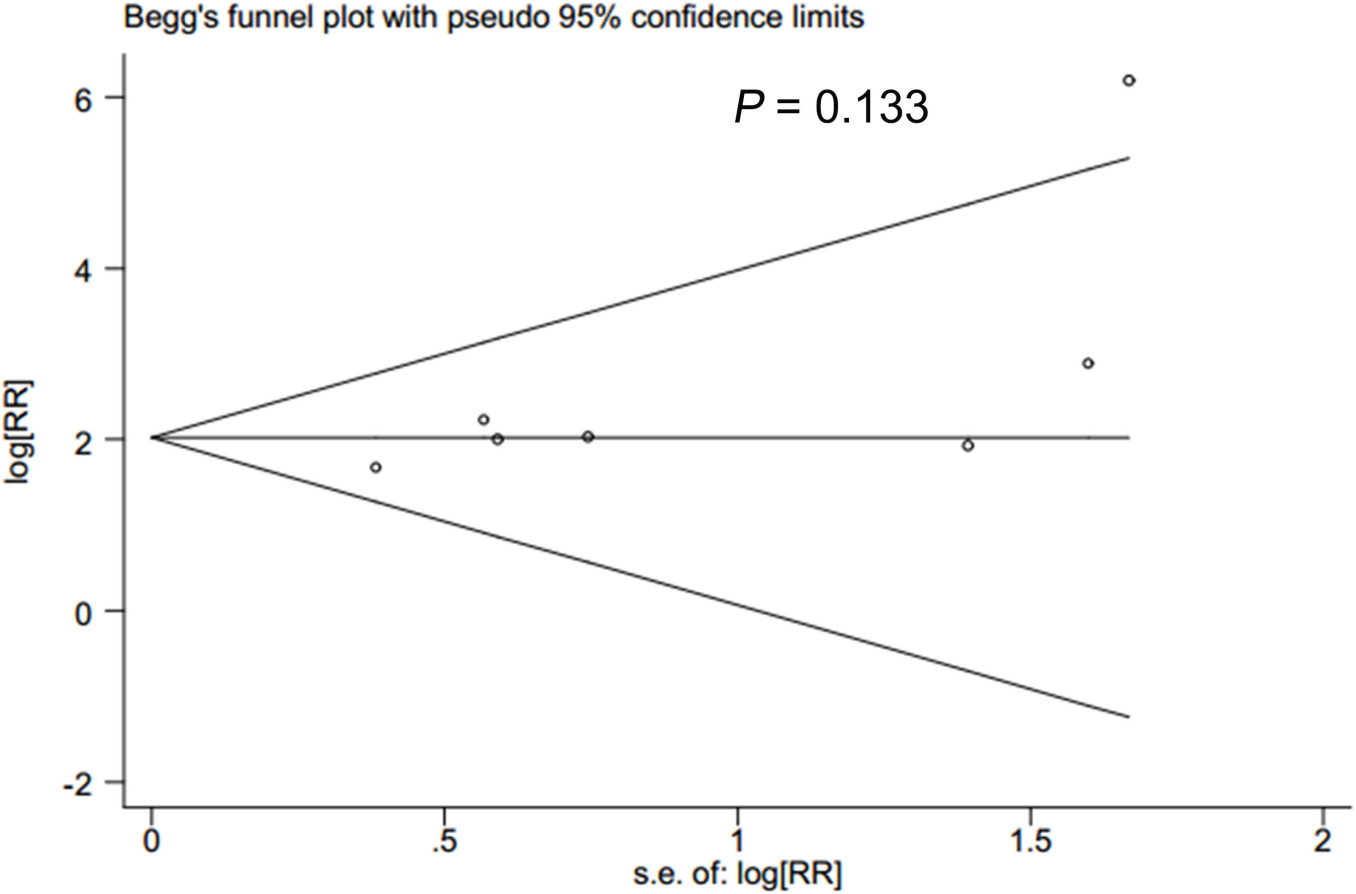
Beggs test to detect publication bias.

In accordance with Begg’s rank correlation analysis and Egger’s linear regression analysis, no publication bias was observed in the studies reviewed (Begg’s, *p* > |z| = 0.133; Egger, *p* > |t| = 0.069) (**Figures 4**, **5**).

## Discussion

Recently, numerous studies (36, 37) had speculated the relationship between CRPC and the positive expression of AR-V7. In this study, we summarized the evidence of the association between CRPC and positive AR-V7 expression. Based on data from the seven included studies, the present study suggested that CRPC patients have a significantly increased positive expression of AR-V7 than the individuals with HSPC. The results derived from this study were consistent with several previous clinical studies (23–25), which demonstrated that the expression of AR-V7 protein was more frequently identified in men with CRPC compared to those with HSPC. Furthermore, we also examined the impacts of potential confounders on our findings by performing the subgroup analyses. A stronger association was detected in RISH measurement in American patients, and those studies were published before 2011 (all *p* < 0.001). Based on the sensitivity analyses, the positive association between CRPC and AR-V7 remained significant in nearly all of the included studies.

A majority of tumors eventually progress to CRPC after a period of an initial response to the ADT regimen (38). The underlying mechanism for the development and progression of CRPC is the expression of AR-Vs, particularly AR-V7, which is a popular area of related research. In ligand-free forms of AR-V7, ligand-binding domains are absent, but transcriptional element binding domains are preserved, facilitating intracellular AR signaling even in the absence of androgens or antiandrogens (39). It is capable of translocating into nuclei and binding AR-responsive elements without ligand interaction and regulating gene transcription (32).

Accumulating evidence has demonstrated that there is a correlation between the level of AR-V7 and resistance to both abiraterone and enzalutamide, as well as poor survival in CRPC (40). In a previous study developed by Seitz et al. (41), 85 CRPC patients were under abiraterone (n = 56) or enzalutamide (n = 29) treatments. High AR-V7 expression levels were associated with shorter prostate-specific antigen (PSA)–progression-free survival (PSA-PFS) (median, 2.4 *vs.* 3.7 months; *p* < 0.001), shorter clinical progression-free survival (median, 2.7 *vs.* 5.5 months; *p* < 0.001), and shorter overall survival (median, 4.0 *vs.* 13.9 months; *p* < 0.001). Moreover, Todenhöfer et al. (42) found that AR-V7 transcripts in peripheral blood were significantly associated with a shorter median PSA-PFS (0.7 *vs.* 4.0 months, *p* < 0.001) and median overall survival (5.5 *vs.* 22.1 months, *p* < 0.001) in CRPC patients treated with abiraterone. This association was confirmed in another clinical study that demonstrated that patients with positive expression of AR-V7 had a poor prognosis than those with negative AR-V7 expression in CRPC treatment with either enzalutamide or abiraterone (43).

Preclinical studies have shown that expression of AR-V7 protein might be a treatment-specific biomarker in CRPC (44). AR-V7-positive patients would benefit from taxanes more than those managed with androgen receptor signaling inhibitors (ARSIs). Antonarakis et al. (45) reported that PSA responses were higher in AR-V7-positive CRPC patients. Moreover, PSA-PFS and clinical and/or radiographic PFS were significantly longer in taxane-treated than those under enzalutamide or abiraterone treatments. However, the outcomes turned out to be non-significant different when the therapy type was changed to AR-V7-negative CRPC. Similar to their results, Scher et al. (46) observed that circulating tumor cell expression of AR-V7 protein in CRPC patients was correlated with superior survival on taxane therapy over ARSI-directed therapy. This evidence suggested the presence of AR-V7 was associated with a better clinical outcome for patients treated with taxanes when compared to those under enzalutamide or abiraterone therapies. Based on the above studies, detecting the AR-V7 expression might be useful to serve as a therapeutic biomarker in patients with CRPC.

In this study, the AR-V7 expression in CRPC patients was found to be significantly ascending as compared with that of patients with HSPC. Moreover, on the basis of the findings in previous studies, the AR-V7 expression might be correlated to resistance to abiraterone and enzalutamide treatment but not taxane chemotherapy. Thus, CRPC patients with positive expression of AR-V7 are recommended to be treated with taxane rather than ARS inhibitors. Accordingly, the AR-V7 status should be evaluated when managing patients with CRPC in clinical practice.

It was speculated that the possible mechanism of progression from HSPC (also known as castration-sensitive prostate cancer (CSPC)) to CRPC is the existence of AR splice variants. AR-V1 was the most common AR-V in hormone-naïve PCa, while AR-V7 was the most common during ADT and in the CRPC stage (47). However, several other variants also have been found to play roles in this action. AR-Vs can be categorized into the following four groups depending on their nuclear localization ability: ligand stimulated in a similar manner to canonical full-length AR (AR-FL) (i.e., AR-23), constitutively active (i.e., AR-V3, 4, 7, and 12), conditionally active (i.e., AR45, AR-V1, and 9), and inactive (i.e., AR-V13, 14, and AR8) (48, 49). For example, in addition to AR-V7, AR-V3, AR-V7, and AR-V9 were also found to be co-expressed in the metastases of CRPC (50). Interestingly, the mRNA of expression of these variants (including AR-V7) was also detected in benign prostatic hyperplasia (BPH) and hormone-naïve primary tumors with lower abundance and frequency (51). De Laere et al. assessed 30 circulating tumor cell (CTC) samples from 26 metastatic CRPC (mCRPC) patients, and 15/26 (57.7%) patients were AR-V-positive with AR-V7 being the most frequently detected variant (12/15, 80%), followed by AR-V3 (11/15, 73%), AR45 (10/15, 67%), AR-V9 (6/15, 40%), AR-V1 (5/15, 30%), AR-V2 (3/15, 20%), and AR-V5 (3/15, 20%) (52). However, a subsequent study (53) developed by To et al. showed that AR-V7 or AR-V9 expression does not predict outcomes in mCRPC patients receiving abiraterone or enzalutamide by conducting a whole blood assay. Nevertheless, based on outcomes from the majority of the previous relevant studies and the meta-analysis of our, AR-V7 was found to be associated with the development and progression of CRPC. At present, most investigators are focusing on AR-V7, but the clinical importance of the detection of other AR-Vs in CRPC is largely unknown, and it deserves further research. According to the above evidence, the specific role of AR-V7 in CRPC is still controversial among different studies. In addition to AR-V7, the expression of several other variants should also be evaluated in both blood and tissues when making a decision on CRPC patients.

In addition to distinct demographic characteristics (i.e., race, sample size, and age), different disease states, and the diverse treatments of CRPC or CSPC, it should be known that various detection methods and measurements for assessing the AR-V7 might also play a role in the outcomes among different studies. For example, Li and colleagues (54) showed that 21% (64/310) of the patients with metastatic CSPC had positive AR-V7 immunohistochemical staining on diagnostic biopsies using clone EPR15656 from Abcam. However, a more recent study (4) reported by Sowalsky et al. demonstrated that AR-V7 mRNA and protein abundance was low in CSPC prior to treatment using robustly validated assays applying both IHC and RNA sequencing-based approaches. The teams previously showed that AR-V7 protein was rarely expressed (<1%) in primary PC but is frequently detected (75% of cases) following androgen deprivation therapy (55). The authors, as have others, also demonstrated that AR-V7 mRNA was commonly identified in benign prostate and primary prostate cancer tissues (4, 51). At present, the majority of the studies were conducted in the metastatic CRPC setting. Our meta-analysis has confirmed the positive relationship between a high level of AR-V7 and the risk of CRPC. However, the clinical importance of AR-V7 detection in HSPC is rarely reported. In HSPC/CSPC research, Li et al. and Sowalsky et al. presented conflicting results about the expression of AR-V7 in CSPC. The following factors may cause this inconsistency: 1) the participants: Li et al. investigated Chinese metastatic HSPC (mHSPC) patients receiving ADT, while Sowalsky et al. investigated US and UK HSPC patients with high-risk localized prostate cancer treated with ADT plus enzalutamide prior to surgery (that is, non-metastatic). 2) The antibody: the AR-V7 antibody used in their studies was different (Li used clone EPR15656 from Abcam; Sowalsky used clone RM7 from RevMAb and Abcam clone EPR15656). 3) The assessment methods for IHC: diverse dilution rate (data were not available) and the cutoff were used for defining “positive or negative” samples. Thus, in the future, evidence for the potential link between AR-V7 and CSPC development may be enhanced if the detection methods are under the same condition. In the CRPC setting, the positive association between AR-V7 and CRPC seems to be a little controversial.

The relationship between AR-V7 and CRPC has been extensively investigated, but whether AR-V7 itself or the ratio of full-length (FL) AR and AR-V7 plays a key role in CRPC remains to be resolved. It is common for patients with CRPC to co-express the full-length receptor and spliced variant AR-V7 (56). After hormone-sensitive PCa progression into CRPC, the expression of full-length AR and AR-V7 increases. AR splice variants exert activating effects in DNA repair genes similar to full-length androgen receptors. However, even in the absence of AR-FL, AR splice variants can provide the necessary transcriptional support for DNA repair genes. AR-Vs have mRNA sequences that are structurally different from the canonical full-length AR. The ARV-7 preferentially leads to the expression of cell cycle regulatory genes, while the full-length AR represses that program and favors instead genes related to metabolism, differentiation, and macromolecular synthesis. Blocking AR-FL using antiandrogens has been shown to retain AR-V activity (47). It was reported that the antibody used for the detection of AR-V7 has high specificity with no cross-reaction to full-length AR (57). In the study of Li et al., mHSPC patients were AR-FL-positive, and 64 (21%) were AR-V7-positive (54).

However, it was reported that AR-V7 could activate target genes irrespective of AR-FL, leading to the development and growth of prostate cancer under low androgen levels (58). AR-V7 are already located at a high fraction in the nuclei of primary PCA cells, while AR-FL remains cytoplasmatic in the absence of activating ligands (59). AR-FL and many AR-Vs may share common chromatin binding sites, genomic binding sites, and transcriptional programs specific to AR-V7. Although AR-V7 co-exists with AR-FL, genomic functions mediated by AR-V7 do not require the presence of AR-FL. AR-V7 expression was found to be lower than AR-FL in CRPC clinical samples (33). AR-V7 is negatively regulated by androgen signaling mediated by AR-FL (60). Detection of AR-V7 indicates the resistance of CRPC to AR signaling inhibitors (43). It was reported that dihydrotestosterone treatment results in a greater decrease in the AR-V7 expression than AR-FL, indicating a mechanism that preferentially reduces the expression of AR-V7 (58). It was also reported that AR-V7 and the AR-V7/AR-FL ratio increase as the disease progresses from CSPC to CRPC (18). Moreover, high nuclear AR-V7 expression and high nuclear AR-V7/AR-FL ratio were associated with a shorter biochemical recurrence-free survival of PCa (59). Among the CRPC patients before their treatment with abiraterone or enzalutamide, positive AR-V7 detection, but not higher AR-FL was significantly associated with shorter PSA-PFS (35). Based on this evidence, both AR-V7 itself and the AR-V7/AR-FL ratio play important roles in CRPC development, but AR-V7 plays a central functional role in this action. However, studies on the relationship between the AR−V7/AR−FL ratio and CRPC are insufficient; whether AR-V7/AR-FL forms heterodimers under low androgen conditions needs further investigation.

Our study is a high-quality cumulative analysis that was conducted following the PRISMA statement. However, some non-negligible limitations should be acknowledged when interpreting the outcomes derived from this study. First, heterogeneity was unavoidable among different studies, even though the potential causes of heterogeneity were identified by sensitivity analysis and subgroup analyses. The effects of small sample studies might be problematic in these meta-analyses, which resulted in the exaggeration of the summary estimates. Second, the various methods of assessment for AR-V7 among studies might also be one of the limitations of this study. Different kinds of assessments for AR-V7 may have a huge impact on the pooled analysis. Therefore, diverse measurements for detecting the AR-V7 expression might be another source of heterogeneity.

## Conclusion

Our study demonstrated that the positive expression of AR-V7 was significantly higher in patients with CRPC than that in those with HSPC. To improve clinical prognostic, the level of AR-V7 should be evaluated when clinicians determined the preferred treatments for CRPC. However, the present evidence was based on retrospective clinical trials with limited included studies. Therefore, further high-quality, large sample size and multicenter cohort studies are still warranted to validate our findings. At present, the predictive effects of the AR-V7 expression in CSPC/HSPC are controversial among studies, and this needs to be addressed in the near future.

## Data availability statement

The original contributions presented in the study are included in the article/**Supplementary Material**. Further inquiries can be directed to the corresponding author.

## Author contributions

ShaZ: project development and manuscript writing. JL and MS: data collection. XL: data analysis. LZ and ShiZ: manuscript editing. All authors contributed to the article and approved the submitted version.
