# Supplementary material for: The positive relationship between androgen receptor splice variant-7 expression and the risk of castration-resistant prostate cancer: A cumulative analysis
Source: Front Oncol. 2023 Feb 14;13:1053111. doi: 10.3389/fonc.2023.1053111 (PMC9972874; doi:10.3389/fonc.2023.1053111)
Supplement: Supplementary file 1 [file Table_1.doc]

**Table S1**. The methodological quality of the cross-sectional study.

| Item | Welti  et al | Zhang  et al | Saylor  et al | Saylor et al | Qu  et al | Hornberg et al | Hu  et al | Zhu  et al |
| --- | --- | --- | --- | --- | --- | --- | --- | --- |
| 1) Define the source of information (survey, record review) | Yes | Yes | Yes | Yes | Yes | Yes | Yes | Yes |
| 2) List inclusion and exclusion criteria for exposed and unexposed subjects (cases and controls) or refer to previous publications | Yes | Yes | Yes | Yes | Yes | Yes | Yes | Yes |
| 3) Indicate time period used for identifying patients | Yes | Yes | Yes | Yes | Yes | Yes | Yes | Yes |
| 4) Indicate whether or not subjects were consecutive if not population-based | Yes | Yes | Yes | Yes | Yes | Yes | Yes | Yes |
| 5) Indicate if evaluators of subjective components of study were masked to other aspects of the status of the participants | No | No | No | No | No | No | No | No |
| 6) Describe any assessments undertaken for quality assurance purposes (e.g., test/retest of primary outcome measurements) | Yes | Yes | Yes | Yes | Yes | Yes | Yes | Yes |
| 7) Explain any patient exclusions from analysis | Yes | Yes | Yes | Yes | Yes | Yes | No | Yes |
| 8) Describe how confounding was assessed and/or controlled. | Yes | Yes | Yes | No | No | No | Yes | No |
| 9) If applicable, explain how missing data were handled in the analysis | Yes | Yes | Yes | Yes | Yes | Yes | Yes | Yes |
| 10) Summarize patient response rates and completeness of data collection | Yes | Yes | Yes | Ye | Ye | Ye | Ye | Ye |
| 11) Clarify what follow-up, if any, was expected and the percentage of patients for which incomplete data or follow-up was obtained | No | No | No | No | No | No | No | No |
